# Negative Affect Reduces Performance in Implicit Sequence Learning

**DOI:** 10.1371/journal.pone.0054693

**Published:** 2013-01-22

**Authors:** Junchen Shang, Qiufang Fu, Zoltan Dienes, Can Shao, Xiaolan Fu

**Affiliations:** 1 State Key Laboratory of Brain and Cognitive Science, Institute of Psychology, Chinese Academy of Sciences, Beijing, China; 2 School of Psychology, Beijing Normal University, Beijing, China; 3 Sackler Centre for Consciousness Science and School of Psychology, University of Sussex, Brighton, United Kingdom; Centre national de la recherche scientifique, France

## Abstract

**Background:**

It is well documented that positive rather than negative moods encourage integrative processing of conscious information. However, the extent to which implicit or unconscious learning can be influenced by affective states remains unclear.

**Methodology/Principal Findings:**

A Serial Reaction Time (SRT) task with sequence structures requiring integration over past trials was adopted to examine the effect of affective states on implicit learning. Music was used to induce and maintain positive and negative affective states. The present study showed that participants in negative rather than positive states learned less of the regularity. Moreover, the knowledge was shown by a Bayesian analysis to be largely unconscious as participants were poor at recognizing the regularity.

**Conclusions/Significance:**

The results demonstrated that negative rather than positive affect inhibited implicit learning of complex structures. Our findings help to understand the effects of affective states on unconscious or implicit processing.

## Introduction

Mood in part consists of a systematic change in how we think, colouring how the world appears. Mood itself also seems to constitute information about how we should think. For example, a transient positive mood may indicate our processing of the environment is going well, our theories about the world are adequate, and an emphasis on top down processing is appropriate – and a transient negative mood may indicate the opposite. Indeed, the affect-as-information hypothesis assumes that people in negative moods tend to focus on bottom-up and item-specific processing, whereas people in positive moods are likely to engage in top down and relational processing [1]–[4]. Consistent with the theory, research indicates that positive rather than negative affect is linked to more relational processing. For example, people in induced transient happy rather than sad moods are more likely to use stereotypes in judging the personality of a story character [5], to engage in more category-level processing in memorizing lists of words [6], and to falsely recall non-presented words highly associated with presented words [3]. Relatedly, affective states are associated with changes in attention [7]–[9]. Normally, negative affect narrows attention [10], all the better to process details, and positive affect broadens the scope of attention [11], all the better to see the bigger picture.

Although the effect of mood on conscious cognition is well established [1]–[4], the effect of mood on unconscious processes, especially on more complex forms of implicit learning, has not been well addressed. Implicit learning is the acquisition of unconscious knowledge about the structure of an environment [12]–[14]. Implicit learning produces a tacit knowledge base, which can be acquired independently of intentional efforts to learn and can be used implicitly to make accurate decisions about novel stimulus circumstances [15]. Given claims that explicit and implicit (i.e. conscious and unconscious) learning rely on separate cognitive systems ([15]–[17]; contrast [18]), the generality of the effect of mood on cognition has not been established. Specifically, the extent to which the acquisition of unconscious knowledge can be influenced by affective states remains unclear.

The serial reaction time (SRT) task has been one of the most widely used tasks in the study of implicit learning. In a typical SRT task, a stimulus appears at one of several locations on a computer screen and participants are told to press the corresponding key according to the stimulus location shown on the screen, i.e. a choice reaction task. Unbeknownst to them, the order of the stimuli follows a repeating or structured sequence. People are faster to respond when the sequence is structured similarly to the training phase rather than when the sequence is switched from the training phase, indicating learning of the sequential structure. Moreover, such learning occurs even when people deny that there was a sequence, cannot freely report it, or cannot control its generation, indicating the knowledge is (largely) unconscious [19]–[26]. Given people acquire knowledge of relations between stimuli in the SRT task, and given mood affects relational processing, we assume that there should be an effect of mood on implicit learning.

To our knowledge, the first study using the SRT task that is relevant to mood effects, examined the relationship between implicit sequence learning and neuropsychological test performance in depressive patients [27], [28]. Patients, compared to control subjects, showed diminished learning effects which were associated with higher self-reported levels of mood disturbances and higher trait anxiety. While the results are consistent with negative mood reducing performance in implicit sequence learning, depression is associated with various processing deficits. Thus, the results do not directly indicate whether or not implicit learning is influenced by transient mild negative mood states.

Recently, a study [29] found that a negative mood *improved* implicit learning on the Artificial Grammar Learning (AGL) task, but did not detectably influence the SRT task in healthy participants. But it is not clear that just because negative mood enhanced implicit learning on the AGL task that any clear prediction can be made for the effect of mood on the SRT task used, partly because these tasks involve learning different structures. A key component of the knowledge acquired in the AGL task is bigrams, i.e. a chunk of two consecutive letters, such as ‘MT’ (e.g., [30]). A bigram is the simplest sequential relation a person could learn - the relation of one item following another. The SRT task used a more complex structure, a so-called second-order conditional (SOC) sequence, such as 3-2-4-1-2-1-3-4-2-3-1-4 in which two previous elements need to be taken into account in order to predict the next element. In the sequence, after a ‘2’, each of ‘1’, ‘3’, and ‘4’ occurs equally often. But after ‘3–2’, the next element must be ‘4’. A triplet (such as 3-2-4) is a more complex relation than a bigram, requiring two rather than previous sequence elements for accurate prediction.

The effect of mood on learning may well depend on the relational complexity of a task, especially the amount of integrative processing required. [29] did not obtain significant effects of mood on the SRT task, which might be evidence against the theory that mood affects complex learning in the SRT task or it might reflect simple insensitivity of the data to pick up real effects [32]. As [29] did not report raw learning scores, but a fairly complex derived measure over the last three of six blocks, it is hard to assess the sensitivity of its null result. In the absence of an assessment of its sensitivity, nothing can be concluded about whether mood has an effect on the complex implicit knowledge typically acquired in the SRT task. Given the complexity of structures in the SRT task, negative mood, in contrast to facilitating learning on the AGL task, may even reduce implicit learning on the SRT task. The relevance of relational complexity on mood effects in implicit learning was shown by [31]. On an implicit probability learning task (predicting whether a square will appear on either the left or right of the screen), it was found that an induced positive rather than negative mood increased integration over more trials into the past for implicitly learnt responses [31].

The aim of the present study was to explore whether mood has an effect on implicit learning of complex structures using the SRT task. People acquire at least some explicit knowledge in the standard SRT task that uses sequences of locations (e.g. [23]). Combining probabilistic sequences with stimulus displays which vary along several dimensions, i.e. location, shape and colour, in a SRT task, discourages explicit learning [33]–[35]. We are specifically interested in implicit rather than explicit learning. Hence, to discourage explicit learning in the SRT task, we adopted a probabilistic sequence with stimulus displays that varied along shape and colour. The probabilistic sequence embedded two regularities: a shape regularity and a colour-shape relation. “Standard” stimuli followed the (shape and colour-shape) regularities with high probability while “deviant” stimuli deviated from one of the regularities with low probability. If people acquired a regularity, they would respond faster to standard rather than deviant stimuli.

Shapes were the target feature. The sequence of shapes followed the complex SOC sequence, i.e. prediction of the incoming shape required integration over the shapes of the previous two trials. Colours were an irrelevant feature. Thus, to increase the potential of the colour-shape sequence to be learnt (despite colours being task irrelevant), it followed a simple FOC sequence, in that only the colour on the single preceding trial was relevant to predict the shape on the next trial. The main aim of including the colour dimension was to reduce the likelihood of explicit learning. As colour was an irrelevant feature, and attention strongly modulates implicit learning [36], [37], we make no strong predictions about the effect of mood on its learning. Thus, the major aim of the present study focused on the effect of mood on learning the shape sequence.

The shape and colour-shape sequences differed not only in SOC vs. FOC but also in the nature of their elements, and in containing only relevant or also irrelevant features, so they cannot be compared straightforwardly. One might predict, based on the arguments made so far about complexity, that any advantage of positive versus negative mood on the SOC (shape) sequence would be less for the FOC (colour-shape) sequence. Negative mood may even improve implicit learning of FOC sequences, as negative mood promoted learning on an AGL task [29]. However, because of other differences between the sequences (especially the irrelevancy of the colours) this prediction is compromised, an issue we take up in the discussion.

Prior research on the effect of mood on implicit learning in the SRT task [29] has not included measurement of conscious awareness. Thus, it is unclear whether it was the acquisition of conscious or unconscious knowledge that was being investigated in prior studies. There is not yet common agreement on how the conscious status of knowledge should be measured (e.g. [18], [38]). However, two approaches are common for establishing the unconscious status of knowledge: First using “objective measures”, by showing that the knowledge expresses itself in a very selective way, e.g. just in reaction times but not in judgments (like recognition) (e.g. [39]); second, using “subjective measures”, by showing that when knowledge is expressed in judgments, the participant is not aware of having the knowledge allowing the judgments (i.e. there is a metacognitive deficit, e.g. [19], [23], [40], [41]).

In the present study, to test whether or not participants were conscious of the learned knowledge, recognition tests were used for each of the shape and colour regularities. Specifically, on each shape or colour test trial, participants were first asked to respond to several targets as in the training phase and then instructed to report whether the shape of the last target followed the shape or colour regularity. Knowledge (shown to exist by RT data) would be unconscious by objective measures if it could not be fully expressed in recognition. A problem with such objective measures is that poor recognition may be produced by poor test sensitivity [13], [18], [42]; thus we will use Bayesian methods to distinguish insensitive data from data providing support for the null hypothesis [43], [44]. If the knowledge is expressed in recognition, subjective measures can be used. As unconscious knowledge may contribute to recognition performance, participants were asked to report the basis of their recognition on each test trial by ticking one of three options: ‘guess or random’ or ‘intuition’, or ‘rules or memory’. Following previous research [45], [46], the responses for “guess” and “intuition” attributions were taken to indicate those cases where structural knowledge was unconscious (as the responses indicate a lack of awareness of structural knowledge), while the responses for “rules” and “memory” attributions were taken to indicate those cases where structural knowledge was conscious (as the responses - on the face of it - indicate awareness of the nature of the structural knowledge). Thus, only when recognition performance with a ‘rules or memory’ attribution is above chance is there evidence of the acquisition of conscious knowledge (see [43], for a review of evidence that the attributions distinguish knowledge types in ways theoretically expected for the distinction between conscious and unconscious knowledge). In sum, the unconscious status of knowledge will be assessed by a two pronged approach of determining if recognition of sequence structure was poor (objective measures) or, when recognition was good, if meta-cognition about that recognition was poor (subjective measures).

## Experiment 1

In order to reduce the likelihood of explicit learning, Experiment 1 used a probabilistic sequence including two regularities with different levels of complexity: a shape regularity following a SOC sequence and a colour-shape relation following a FOC sequence. Colour was not relevant to the participant’s task. While the SRT task typically, but not always, uses varying locations of the stimuli as (one) key feature, with RT to location acting as the main dependent variable, location differs from many other features such as shape or colour by having a motor as well as a perceptual component. That is, learning a location sequence is partly learning an oculomotor sequence. This is not necessarily a problem, but it is cleaner to use two features which are more similar in being more clearly perceptual.

Negative and positive music pieces were used to induce different affective states, which proved to be effective in previous research [11]. The SRT task involves a long training phase. To maintain the induced mood, a short version of the mood induction was repeated after the first half of the training blocks. To check the effect of the mood induction, participants were asked to rate the valence of their moods and the valence of neutral pictures (as suggested by [47]) before and after the music induction.

The major aim of Experiment 1 was to determine the effect of mood on implicitly learning the shape sequence. A secondary aim was to determine if the irrelevant colour-shape sequence was learnt, and thus if there were any effects of mood on its learning.

### Participants

Eighty undergraduate students (40 male, 40 female) took part in the experiment. None of them had previously taken part in any implicit learning experiment. They were randomly assigned to two groups (positive, *n* = 40; negative, *n* = 40). To ensure that only data from participants who performed the SRT task according to the instruction were analyzed, participants were excluded if their error proportions were greater than.15. Data from one participant in the positive group (*M* = .31) and three participants in the negative group (*M* = .24, *SD* = .12) were excluded. All participants were tested between 9 and 11 a.m. or between 2 and 5 p.m., which was counterbalanced between the two groups.

### Ethics Statement

This experimental procedure was approved by the committee for the protection of subjects at the Institute of Psychology, Chinese Academy of Sciences. Written consent for the collection of data and subsequent analysis was obtained from each participant. This procedure was also followed in Experiment 2.

### Apparatus and Materials

**Figure 1:**
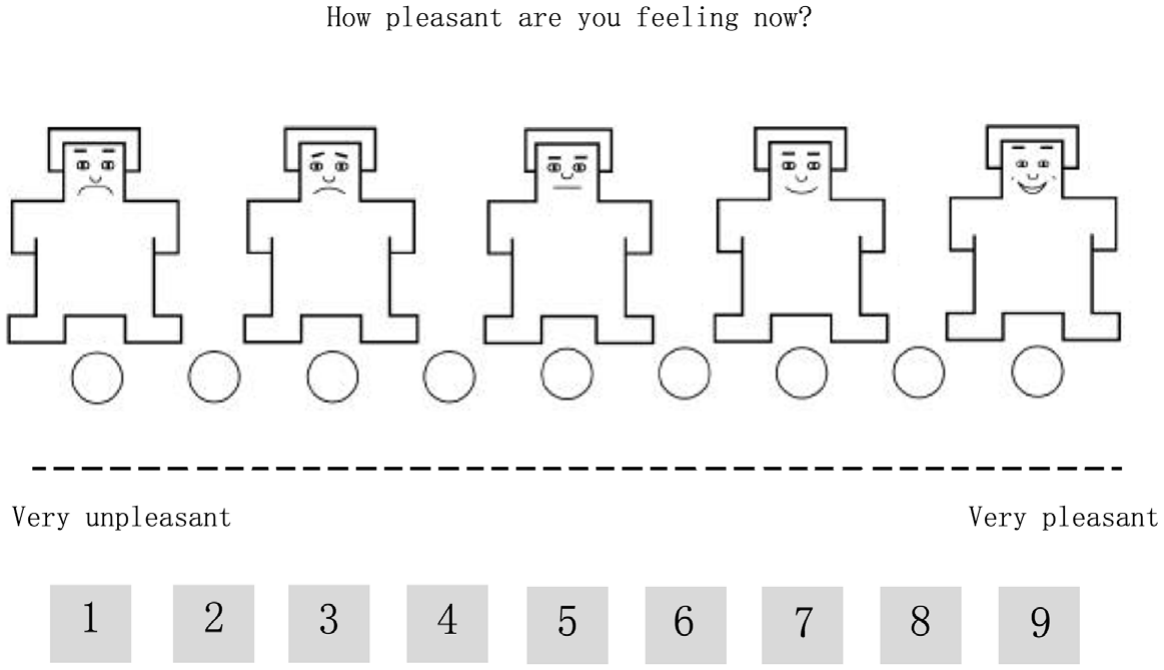
The Self-Assessment Manikin (SAM) Used to Measure the Valence.

The experiment was programmed in E-prime 1.2 and ran on Pentium-compatible PCs. For the positive mood induction, participants were asked to listen to a jazzed-up version of Bach’s *Brandenberg Concerto No.3* (played by Hubert Laws). For the negative mood, participants were instructed to listen to Prokofiev’s *Alexander Nevsky: Russia under the Mongolian Yoke* played at half speed. Two methods were used to check the effect of mood induction throughout the training. First, participants were asked to directly rate the valence of their moods before and after the music induction by using a picture scale, i.e. the Self-Assessment Manikin (SAM) [48]. The SAM is a picture scale which can be directly used to measure the three basic dimensions of emotion: valence, arousal, and dominance [50], [51]. Each dimension is measured on a continuous nine-point scale (ranging from 1 to 9). Only the valence dimension was used in this study. As can be seen from Figure 1, the SAM is a 1-trial procedure each time it is administered and the valence is measured on a continuous nine-point scale, ranging from 1 (very unpleasant) to 5 (neutral) to 9 (very pleasant). Second, 32 neutral pictures from the Chinese Affective Picture System [49] were rated on a similar nine-point scale before and after the music induction and after each training block, in order to assess mood indirectly. According to a normative study [49], the mean valence ratings, arousal ratings, and dominance ratings of the pictures on a nine-point scale were 5.35 (*SD = *0.07), 3.72 (*SD = *0.40), and 5.94 (*SD* = 0.72), respectively, with higher rating indicating more positive or arousing or dominant.

For implicit sequence learning, the display consisted of a stimulus in a square in the center of the computer’s screen against a gray background. The stimuli were squares, triangles, circles, and hearts in different colors. Shapes were the target feature while colors were the task-irrelevant feature. Each shape could be in three of the four different colors including red, green, yellow, and blue, according to our regularity. For example, if green predicts triangle, triangle could be in red, yellow or blue, but could not be in green because two continuous stimuli could not be in the same colour. Thus, there were 12 color-shape combinations. On each trial, a colored shape appeared in the square, which covered a visual angle of approximately 1°.

### Procedure

#### Mood induction and measurement

Participants were first asked to rate the valence of four neutral pictures as practice and rated the valence of two neutral pictures formally on a nine-point scale. Next they rated the valence of their initial moods by using the SAM [48]. Thereafter, they were instructed to listen to the designated music and generate matching thoughts for 10 min. When the music was finished, they were asked to rate the valence of their moods and of two neutral pictures, successively. After each block of the SRT task, participants were also asked to rate the valence of two neutral pictures. A shorter 5-minute version of the mood induction was repeated after the sixth block. Participants were asked to rate the valence of their moods before and after the repeated mood induction. Thus, participants were required to rate the valence of their moods at four time points and the valence of neutral pictures at 14 time points throughout the training phase. Sixteen neutral pictures were randomly assigned to the first eight time points separately for each participant; a different set of twelve neutral pictures was used to randomly assign to the last six time points separately for each participant.

#### The SRT task

**Figure 2:**
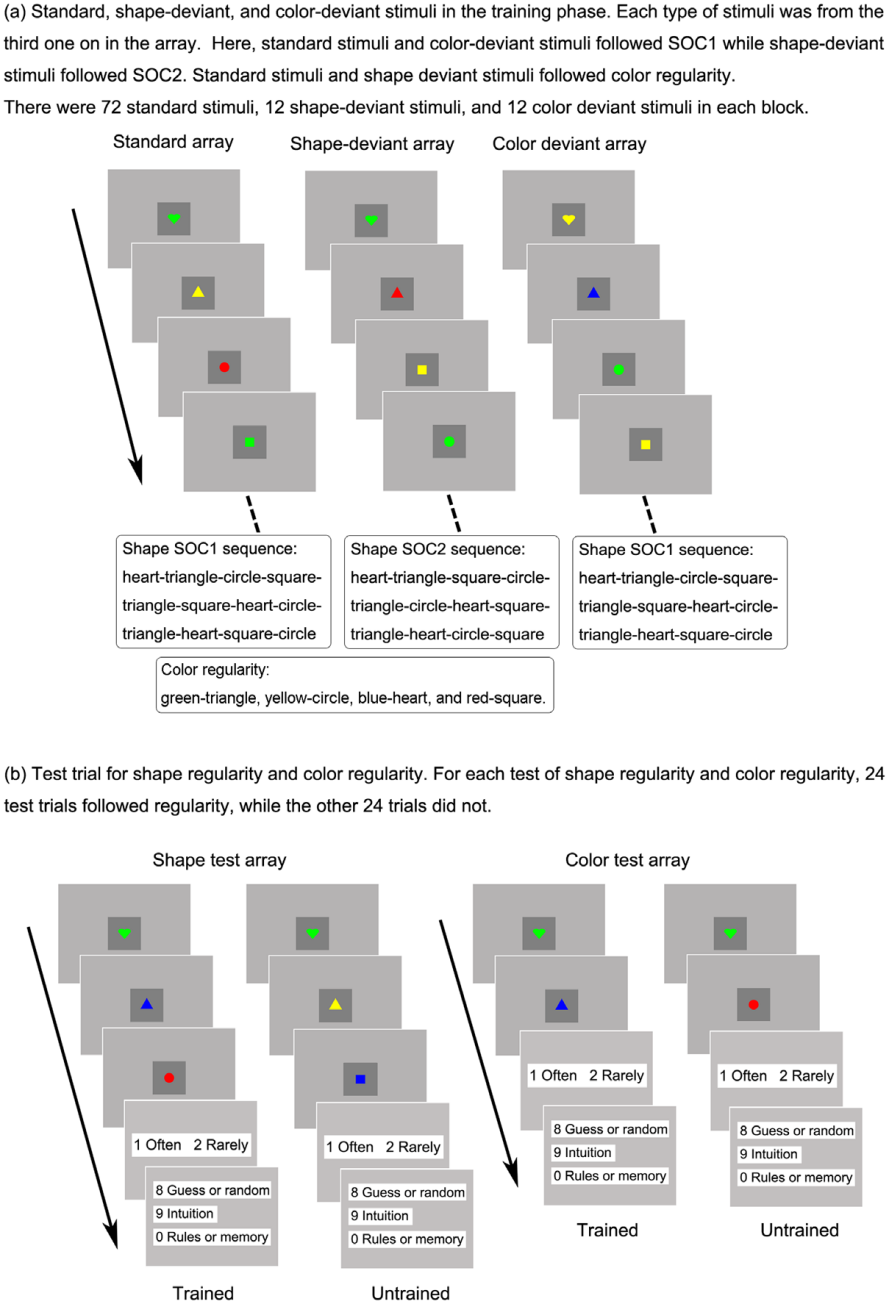
Experimental setup and design in Experiment 1.

Following the mood induction, participants were exposed to the SRT task, which included 12 training blocks. Each block consisted of 98 trials, for a total of 1176 trials. On each trial, participants were instructed to identify the shape of the target as quickly and as accurately as possible by pressing the corresponding key. Keys D, F, J, and K corresponded to the square, triangle, circle, and heart, and were required to be pressed by participants’ left middle and index fingers and right index and middle fingers, respectively. The target was presented on the screen until the correct key was pressed, and the next target appeared immediately (i.e. the response stimulus interval was zero) to reduce the likelihood of explicit learning [21], [22], [33]. Response latencies were measured from the onset of the target to the completion of a correct response. The targets followed two different sequence structures: the shape regularity and the colour regularity. The shape regularity means that the shape of each target was determined by the shapes of the previous two targets. That is, the shape sequence of the targets followed one of the two second-order conditional sequences (SOC1 = heart-triangle-circle-square-triangle-square-heart-circle-triangle-heart-square- circle; SOC2 = heart-triangle-square-circle-triangle-circle-heart-square-triangle-heart-circle-square). The colour regularity means that the shape of the target was determined by the colour of the previous stimulus. That is, the colour-shape sequence of the targets followed the first-order conditional sequences (green-triangle, yellow-circle, blue-heart, and red-square). For example, on the basis of the SOC1 sequence, after a heart and then a triangle, next should be a circle; on the basis of the FOC sequence, after a green shape, the next should be a triangle (see Figure 2a).

In each block, there were three types of targets: standard, shape-deviant, and colour-deviant targets. Standard targets were targets following both the shape and colour regularity, with a probability of 0.75 (i.e., 72 trials in each block). Shape-deviant targets were targets following the colour regularity but not the shape regularity, with a probability of 0.125 (i.e., 12 trials in each block). Colour-deviant targets were targets following the shape regularity but not the colour regularity, with a probability of 0.125 (i.e., 12 trials in each block). Each training block began at a random point in one of the two SOC sequences. It was continued by a sequence of 12 targets of the same type (i.e., standard or deviant), and then transferred to another sequence of 12 targets of the same type. Standard targets could transfer to any type of target but deviant stimuli could only transfer to standard ones. For half of participants, standard targets followed both the SOC1 and FOC regularity, while shape-deviant stimuli followed SOC2 and colour-deviant stimuli changed with equal probability to one of the other three colours; for the other half, standard targets followed the SOC2 and FOC regularity, whereas shape-deviant stimuli followed SOC1 and colour-deviant stimuli changed with equal probability to one of the other three colours. The sequential position of shape-deviant and color-deviant stimuli in each block were counterbalanced during training. Each block of the SRT task took about one and a half minutes. After each block, participants were asked to rate the valence of two neutral pictures and then had at least 20 seconds for a short rest. The training phase took about 30 minutes.

#### Recognition tests

After the SRT task, there were two recognition tests: one for the colour regularity, the other for the shape regularity. At the beginning of the test for colour regularity, participants in each group were informed that the sequence of targets followed a regularity, in which most of shapes were determined by the colour of the previous target (see Figure 2b). On each colour test trial, participants were required to first respond to two targets using the same response keys as for the training phase. Then, they were instructed to report whether the shape of the second target followed the colour of the previous one often (with a probability of about 90%) or rarely (with a probability of about 10%). At the beginning of the test for the shape regularity, participants in each group were informed that the order of targets followed a regularity, in which most of shapes were determined by the shapes of the previous two targets. On each shape test trial, participants were asked to first respond to three targets using the same responses keys as for the training. They were then required to report whether the shape of the third target followed the shapes of the previous two often (with a probability of about 90%) or rarely (with a probability of about 10%). After each test trial, participants were required to report the basis of their judgment by picking one of the following: guess or random, intuition, rules or memory. Participants were provided with definitions taken from [45]. There were 48 test trials for each test, in which half of them followed the corresponding regularity and half of them did not. The irregular color test trials were taken from the color-deviant sequence and the irregular shape test trials were taken from the shape-deviant sequence except that the third target was not predicted by the color of the second one. Before each test, there were four practice test trials. The order of color test and shape test was counterbalanced between participants.

### Results

We will consider the following questions in order: Was the mood induction successful? Then, crucially, did mood influence learning? Finally, were people consciously aware of each regularity?

**Figure 3:**
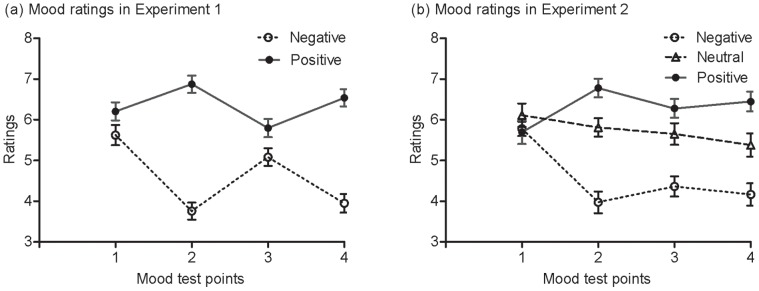
Mood ratings at four time points in Experiments 1 and 2. For Experiment 1, the first time was before mood induction, the second one was immediately after the mood induction, the third one was after six blocks and before the repetition of the short version of mood induction, and the fourth one was immediately after the second mood induction. For Experiment 2, the first time was before mood induction, the second one was immediately after the mood induction, and the third and fourth one was after second and third mood induction. Error bars depict standard errors.

**Was the mood induction successful?** Figure 3 shows mood ratings at four time points in Experiments 1 and 2. The first mood test was before the music induction and the second one was immediately after the music induction. For Experiment 1, to examine whether the mood manipulation was successful, an ANOVA on mood ratings with mood test (first vs. second) as a within-subject variable and group (negative vs. positive) as a between-subjects variable revealed a significant mood test effect, *F* (1, 74) = 6.61, *p*<.05, *η* ***_p_*** ^2^ = .08, a significant group effect, *F* (1, 74) = 75.23, *p*<.001, *η* ***_p_*** ^2^ = .50, and a significant interaction, *F* (1, 74) = 29.52, *p*<.001, *η* ***_p_*** ^2^ = .29. Initially, there was no difference between the negative and positive groups, *t* (74) = −1.76, *p* = .083, *d* = .40, but after the mood induction the ratings in the positive group were much more positive than those in the negative group, *t* (74) = 10.44, *p*<.001, *d* = 2.39. Specifically, negative mood inductions decreased valence ratings, *t* (33) = 5.51, *p*<.001, *d* = .96, while positive inductions increased valence ratings, *t* (37) = −2.11, *p*<.05, *d* = .35, indicating that the mood induction was effective. One-sample *t* tests were used to compare ratings to the neutral mood point for each condition after the mood induction. Participants in the negative group had a valence significantly below neutral, *t* (36) =  −5.92, *p*<.001, *d* = −.99, but participants in the positive group had a valence significantly above neutral, *t* (38) = 8.85, *p*<.001, *d* = 1.44.

The second, third, and fourth mood tests were all after the initial mood induction. To test whether the mood difference persisted through training, an ANOVA with the ratings at the last three mood tests as a within-subject variable and group as a between-subjects variable revealed a significant group effect, *F* (1, 74) = 79.73, *p*<.001, *η* ***_p_*** ^2^ = .52, and a significant interaction of mood tests by group, *F* (1, 74) = 29.55, *p*<.001, *η* ***_p_*** ^2^ = .29. The ratings at the third mood test, i.e. before the repetition of the mood induction, increased in the negative group, *t* (36) =  −4.98, *p*<.001, *d* = −.83, but decreased in the positive group, *t* (38) = 4.26, *p*<.001, *d* = .69, compared with the ratings at the second mood test. However, the ratings at the fourth mood test after the repetition of mood induction decreased in the negative group, *t* (36) = 4.14, *p*<.001, *d* = .69, and increased in the positive group, *t* (38) =  −3.21, *p*<.01, *d* = −.52, compared with the ratings at the third one, indicating the usefulness of the repetition of the mood induction. Importantly, participants in the positive group reported higher valence ratings than those in the negative group in all the last three tests, *t* (74) = 10.44, *p*<.001, *d* = 2.43, *t* (74) = 2.30, *p*<.05, *d* = .53, *t* (74) = 8.41, *p*<.001, *d* = 1.96, confirming that there was a mood difference between the two groups throughout the training phase.

**Figure 4:**
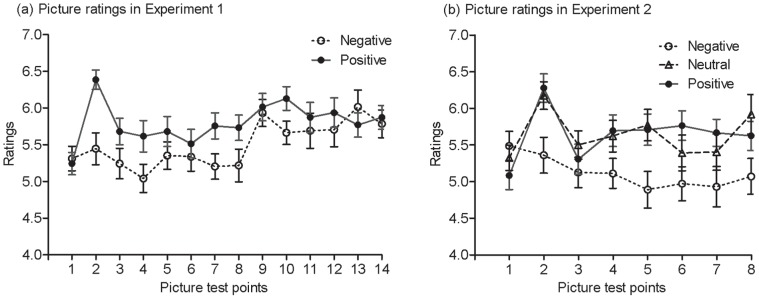
Rating of pictures at fourteen time points in Experiments 1 and 2. The first time was before mood induction and the second one was after the mood induction. Each of the last 12 tests was before each of the 12 training blocks. Error bars depict standard errors.

Figure 4 shows ratings of pictures in Experiments 1 and 2. The pattern of ratings of pictures was similar to that of mood ratings. The first picture rating was before the music induction and the second one was after the music induction. For Experiment 1, to examine whether the mood induction changed the ratings of pictures, an ANOVA with picture tests (first vs. second) as a within-subject variable and group (negative vs. positive) as a between-subjects variable revealed a significant picture test effect, *F* (1, 74) = 15.69, *p*<.001, *η* ***_p_*** ^2^ = .18, a significant group effect, *F* (1, 74) = 6.11, *p*<.05, *η* ***_p_*** ^2^ = .08, and a significant interaction, *F* (1, 74) = 9.75, *p*<.01, *η* ***_p_*** ^2^ = .12. The mood induction increased valence ratings in the positive group, *t* (38) =  −6.21, *p*<.001, *d* = −1.28, but did not significantly decrease valence ratings in the negative group, *t* (36) = .50, *p* = .62. Importantly, at the initial test, there was no difference between the negative and positive groups, *t* (74) = .30, *p* = .77, but higher positive ratings in the positive group than in the negative group after the mood induction, *t* (74) = 3.74, *p*<.001, *d* = .87, revealing that the mood induction successfully changed the ratings of the pictures.

The ratings at the last 13 time points were all after the initial mood induction. To test whether there was a rating difference between negative and positive groups throughout training, an ANOVA with ratings on the picture tests after mood induction (13 levels) as a within-subject variable and group as a between-subjects variable revealed a picture test effect, *F* (12, 888) = 3.74, *p*<.001, *η* ***_p_*** ^2^ = .05, indicating higher positive ratings later on than earlier. Importantly, the main effect of group reached significance, *F* (1, 74) = 4.85, *p*<.05, *η* ***_p_*** ^2^ = .06, but the interaction of group by picture test was not significant, *F* (12, 888) = 1.56, *p* = .10, confirming overall higher positive affect in the positive group than in the negative group. However, as can be seen from Figure 4a, although overall, there were higher positive ratings in the positive group than in the negative group, the rating differences may not be stable over time (despite the non-significance of the interaction). Independent samples *t* tests revealed that there were nonetheless significant rating differences at time points 2, 4, 7, 8 and 10 (*p*s <.05, one-tailed).

**Did mood influence learning?** The mean RTs for all shape-deviant, standard, and colour-deviant trials with correct responses were 772.32 (*SD* = 120.45), 725.92 (*SD* = 114.70), 724.91 (*SD* = 119.02), respectively, for all participants. Trials with RTs greater than 2,000 milliseconds were excluded from analysis because they could have resulted from irrelevant activities such as adjusting glasses etc. There were 1.25% and 1.36% of the trials in the negative and positive groups being removed, respectively.

**Figure 5:**
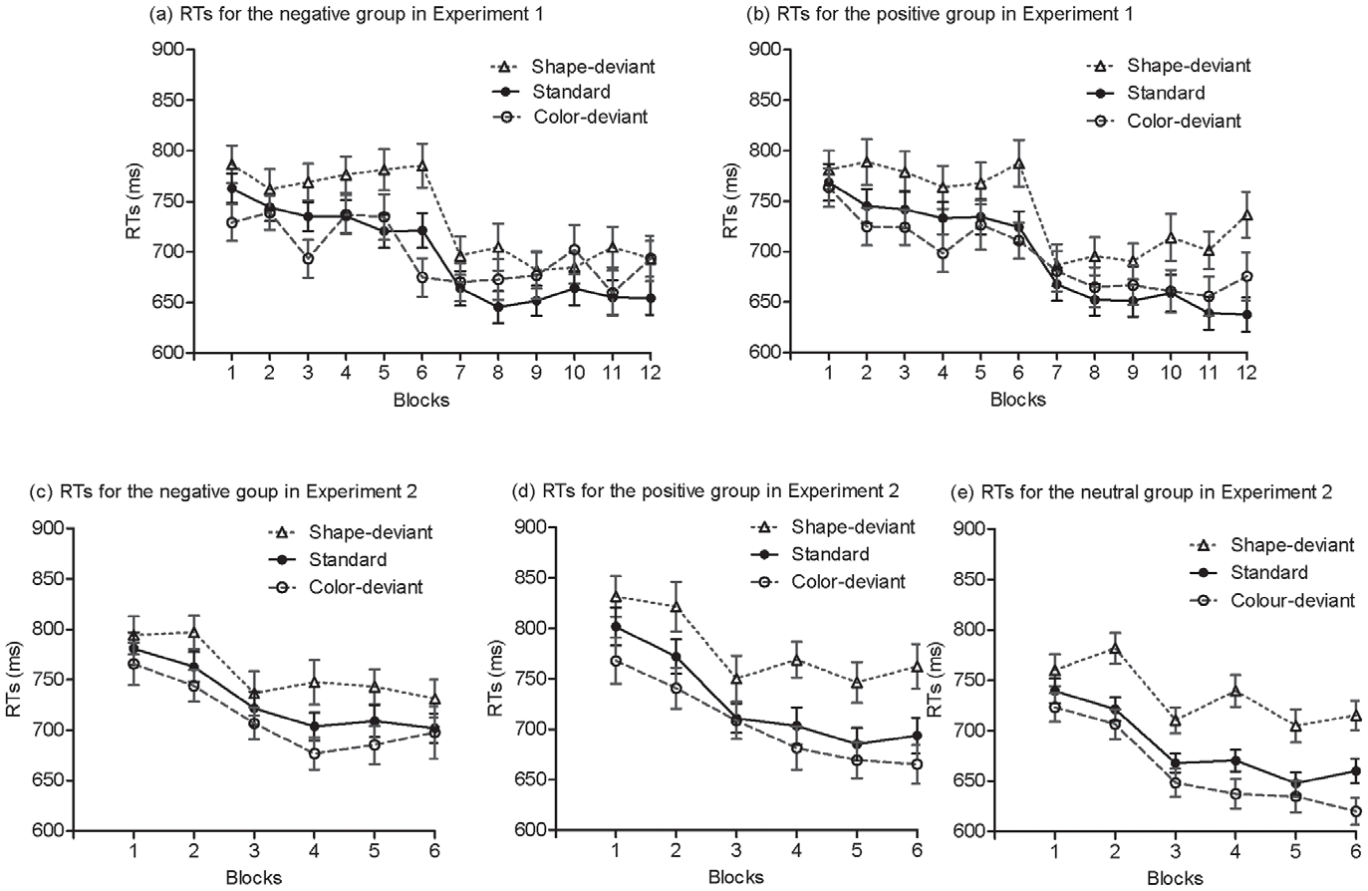
Mean reaction times for shape-deviant, standard, and color-deviant stimuli across training blocks in Experiments 1 and 2. Error bars depict standard errors.

**Figure 6:**
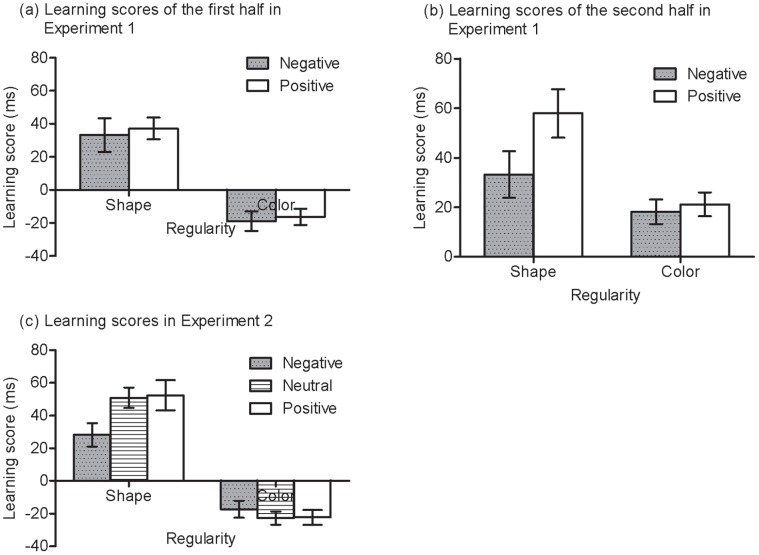
Learning effects for shape regularity and color regularity in Experiments 1 and 2. Error bars depict standard errors.

If participants learned the shape regularity, they would respond to the standard targets faster than the shape-deviant targets; similarly, if participants learned the colour regularity, they would respond to the standard targets faster than the colour-deviant targets. Figure 5 shows the mean RTs obtained over the training phase for each group in Experiments 1 and 2. As can be seen from Figures 5a, there was a sudden drop in RTs from Block 7 after the second mood induction. Participants in each group listened to the designated music for five minutes in the second mood induction in Experiment 1. As the speed up occurred for both standard and deviant stimuli it is unlikely to be consolidation (cf. [52]); but the effect is precisely as predicted by [53] release from reactive inhibition, i.e., a speeding up on a task after a rest. For simplicity, the data were re-expressed as a learning score separately for the shape and colour regularities. Specifically, the learning score for shape was the RTs (reaction time) to shape deviants minus the RT to standards. Similarly, the learning score for colour was the RTs to colour-deviants minus the RTs to standards. The learning score was averaged over the first 6 blocks to obtain a learning score for the first half of training and over the last 6 blocks to obtain a learning score for the second half in Experiment 1. Figure 6 shows these summarized data. If there was no learning, the learning score would be zero.

For Experiment 1, one-sample *t* tests were used to compare learning scores with chance (i.e. zero) for each combination of regularity, experimental half and group. This revealed that there was significant learning of the shape regularity (*t* (36) = 3.93, *p*<.001, *d* = .66, *t* (38) = 5.65, *p*<.001, *d* = .91), but no learning of the colour regularity (*t* (36) = −3.18, *p*<.01, *d* = −.53, *t* (38) = −3.26, *p*<.01, *d* = −.53) in negative and positive groups in the first half of the experiment. However, importantly, both regularities were learnt significantly in both groups (all *p*s <.01) in the last half of the experiment.

To test whether there was an effect of mood on learning the shape regularity, we performed an ANOVA on learning scores for the shape regularity, with group (positive vs. negative) as a between-subject variable, and training half (first vs. second) as a within-subject variable. This revealed only a significant interaction between group and training half, *F* (1, 74) = 5.73, *p*<.05, *η* ***_p_*** ^2^ = .07. The positive group showed similar learning to the negative group in the first half, *t* (74) = .22, *p* = .83, but more learning in the second half, *t* (74) = 1.82, *p*<.05 (one-tailed), *d* = .42. The results suggested that negative rather than positive affect impaired the learning of the complex regularity.

To test whether there was an effect of mood on learning of colour regularity, a similar ANOVA on colour learning scores was used. It revealed only an effect of training half, *F* (1, 74) = 48.40, *p*<.001, *η* ***_p_*** ^2^ = .40, indicating learning. Note that in the first half of training the learning scores were significantly below chance. This arises because colour-shape pairings were not counterbalanced and pre-existing biases determined learning scores in the first half of training, providing a baseline against which to measure learning. For example, in the first block, all participants expressed a significant tendency to respond to standard stimuli slower than irrelevant-deviant stimuli overall (*M* = −21.77, *SE* = 8.26), *t* (75) = −2.64, *p* = .01. However, the direction of the response bias for each of the four colour rules was different. Specifically, people responded to standard stimuli after red and blue significantly faster than the corresponding irrelevant-deviant stimuli (*M* = 72.16, *SE* = 21.26, *M* = 72.00, *SE* = 20.41), *t* (75) = 3.40, *p* = .001, *t* (75) = 3.53, *p* = .001, respectively. Meanwhile, people responded to standard stimuli after green and yellow significantly slower than the corresponding irrelevant-deviant stimuli (*M* = −129.31, *SE* = 17.49, *M* = −101.95, *SE* = 22.02), *t* (75) = −7.40, *p*<.001, *t* (75) = −4.63, *p*<.001, respectively. That is, half of the biases were positive and half of them were negative in direction, just as would be expected, though to get exactly half could not have been guaranteed. It so happens the biases were bigger in some cases than others, and such natural viability is also just as expected. Hence we see an overall initial bias. Indeed, by the end of training, these biases had been over-ridden by learning [54]. Importantly, we did not detect a difference between the two groups in either the first or second half of the training (both *p*s >.66).

We found a difference between positive and negative groups in the second half of training for the shape regularity, but did not for the colour regularity. The latter null result may simply reflect insensitivity rather than the absence of an effect. How big a mood effect might we expect? For the shape regularity, the learning score for the positive group (58 ms) was about twice that as for the negative group (33 ms). Relatedly, normal people performed about twice as well as depressed people on the SRT task [27]. The learning effect for colours was about 20 ms (on average and for negative group), so something like a 20 ms mood effect could be expected, if it existed. We used Baysian analysis to model the predictions of the alternative with a half-normal with a standard deviation of 20 ms, based on the considerations in the text and following recommendations in [32] (Appendix). The Bayes Factor comparing the hypothesis of a mood effect to the null hypothesis of no mood effect was.47. Bayes Factors vary between 0 and infinity with values of less than.33 indicating support for the null hypothesis and values greater than 3 indicating support for the alternative. Values in between indicate data insensitivity (see [32], [55], for explanation of Bayes Factors and free online software). Thus, the results indicated the data were insensitive and no strong conclusions should be drawn from the null result.

**Were people consciously aware of the colour regularity?** If participants consciously learned the colour regularity, they would perform better than chance (i.e. 0.5) in the recognition for colour regularity, especially when the recognition was based on rules or memory. Table 1 shows accuracy rates in the recognition test of colour regularity in Experiments 1 and 2. Overall, participants in both positive and negative groups performed at chance in the recognition test of the colour regularity, *t* (38) =  −.05, *p* = .96, *t* (36) = −1.08, *p* = .30, respectively. Due to the overall chance-level performance, we did not further analyze the proportions (see Table 2) or accuracy rates for each attribution in the color test (i.e. guess or random, intuition, rules or memory).

**Table 1:** Accuracy Rates in the Recognition Test of Color Regularity in Experiments 1 and 2.

		Guess orrandom	Intuition	Rules ormemory	Total
Experiment 1	Negative	.50 (.05)	.49 (.02)	.53 (.03)	.50 (.01)
	Positive	.50 (.03)	.51 (.03)	.49 (.02)	.49 (.01)
Experiment 2	Negative	.49 (.05)	.56 (.03)	.46 (.03)	.49 (.01)
	Neutral	.46 (.04)	.44 (.02)	.48 (.04)	.48 (.01)
	Positive	.45 (.05)	.55 (.03)	.48 (.02)	.50 (.01)

Notes:Standard Errors in Brackets.

**Table 2:** Proportions of Each Attribution in the Recognition Test of Color Regularity in Experiments 1 and 2.

		Guess or random	Intuition	Rules or memory
Experiment 1	Negative	.22 (.04)	.46 (.04)	.32 (.05)
	Positive	.23 (.04)	.30 (.04)	.47 (.05)
Experiment 2	Negative	.21 (.04)	.39 (.05)	.40 (.06)
	Neutral	.24 (.04)	.45 (.05)	.31 (.04)
	Positive	.19 (.04)	.40 (.04)	.40 (.05)

Notes: Standard Errors in Brackets.

To interpret this null result, the expected recognition performance that would obtain if the knowledge were all conscious needs to be estimated. The colour regularity consisted of four rules: green-triangle, yellow-circle, blue-heart, and red-square. We calculated and compared the RTs for standard and color-deviant targets corresponding for each of them in the second half of the training. On the basis of the comparison results, two rules were learned significantly (*t* (36) = 4.17, *p*<.001, *d* = .70, *t* (36) = 1.77, *p*<.05 (one tailed), *d* = .30, for negative group; *t* (38) = 2.31, *p*<.05, *d* = .37, *t* (38) = 2.25, *p*<.05, *d* = .37, for positive groups) while one was not learned (*ps* >.13 for both groups) and one was learned the wrong way (*t* (36) = −2.05, *p*<.05, *d* = .34, *t* (38) = −2.14, *p*<.05, *d* = .35, respectively) by participants in both groups. If this knowledge were completely conscious, people would recognize correctly the two rules and recognize incorrectly one and guess the other one, producing an expected recognition performance of (2*1+1*.5+1*0)/4 = 62.5%. The upper limit of the confidence interval on recognition performance was 52% and 51%, for positive and negative groups respectively, substantially different from 62.5%, suggesting that neither group could apply the knowledge they acquired in the training phase to the recognition test. That is, their knowledge about the color regularity was unconscious or implicit.

Given we have identified all the learned triplets, 62.5% defines strictly just an upper limit expected on the theory that all knowledge is conscious because there could be random noise in any given recognition judgment, for example as postulated by [56]. Given this noise could be any amount from 0% to 100% of the recognition signal, the expected recognition performance could be any value from 50% to 62.5% with equal probability assuming that all knowledge was conscious. The null hypothesis cannot be supported over the theory that recognition could be uniformly any value from 50% to 62.5% using conventional statistics (*p*_values, power). But Bayesian statistics are capable of evaluating the support for such theories [55]. A Bayes Factor testing this alternative theory that all knowledge was conscious against the null hypothesis that no knowledge was conscious was.10 for the negative group and.05 for the positive group, constituting very strong evidence against the theory and in favor of the null hypothesis. See [32], [55] for further discussion of the use of Bayes Factors.

**Were people consciously aware of the shape regularity?** If participants consciously learned the shape regularity, they would perform better than chance level (i.e.,.50) in the recognition test for the shape regularity, especially when the recognition was based on rules or memory. Table 3 shows accuracy rates in the recognition test of shape regularity in Experiments 1 and 2. For Experiment 1, participants in both positive and negative groups performed significantly above chance in the recognition test of the shape regularity, *t* (38) = 2.58, *p*<.05, *d* = .35, *t* (36) = 2.15, *p*<.05, *d* = .41, respectively.

**Table 3:** Accuracy Rates in the Recognition Test of Shape Regularity in Experiments 1 and 2.

		Guess or random	Intuition	Rules or memory	Total
Experiment 1	Negative	.56 (.05)	.48 (.02)	.54 (.02)	.52 (.01)*
	Positive	.53 (.05)	.50 (.03)	.56 (.02)*	.53 (.01)*
Experiment 2	Negative	.49 (.04)	.51 (.03)	.52 (.03)	.51 (.01)
	Neutral	.51 (.04)	.49 (.02)	.61 (.03)*	.52 (.01)
	Positive	.43 (.05)	.53 (.03)	.56 (.03)*	.53 (.01)*

Notes: *p<.05. Standard Errors in Brackets.

Table 4 shows proportions of each attribution in the recognition test of shape regularity in Experiments 1 and 2. As can be seen from Table 4, participants based their judgment on both conscious and unconscious knowledge. For Experiment 1, an ANOVA on proportions with attribution (guess or random vs. intuition vs. rules or memory) as a within-subject variable and group (negative vs. positive) as a between-subjects variable revealed only a significant attribution effect, *F* (2, 148) = 18.67, *p*<.001, *η* ***_p_*** ^2^ = .20. All participants attributed to rules and memory more than intuition, *t* (75) = 2.19, *p*<.05, *d* = .25, and attributed more to intuition than guess and random, *t* (75) = 4.27, *p*<.001, *d* = .49. Recognition accuracy was compared with chance for each combination of mood condition and attribution type (see Table 3). Participants in the positive group performed significantly above chance when they gave rules and memory attributions, *t* (37) = 3.00, *p*<.01, *d* = .49, but not for the other two attributions (both *p*s >.47), suggesting participants in the positive group acquired some conscious knowledge. Participants in the negative group did not perform significantly different from chance in any of the three attributions (all *p*s >.11), revealing no evidence for conscious knowledge in the negative group. The significant result for the positive group was for rules or memory, with a recognition of.56 (*SE* = .02). Thus, if there was above-chance recognition performance for the negative group, a reasonable expectation might be represented by a half normal with a standard deviation of.05, i.e., recognition memory is very likely to be in the interval.50–.60, if it exists. The Bayes Factor was 1.63 for guess or random,.17 for intuition, and 1.98 for rules or memory, suggesting insensitive evidence that knowledge was conscious in the negative group.

**Table 4:** Proportions of Each Attribution in the Recognition Test of Shape Regularity in Experiments 1 and 2.

		Guess or random	Intuition	Rules or memory
Experiment 1	Negative	.18 (.03)	.38 (.04)	.44 (.04)
	Positive	.20 (.03)	.32 (.03)	.48 (.04)
Experiment 2	Negative	.16 (.03)	.42 (.05)	.43 (.06)
	Neutral	.19 (.03)	.48 (.05)	.33 (.05)
	Positive	.18 (.03)	.42 (.04)	.40 (.04)

Notes: Standard Errors in Brackets.

Again to provide a scale of how much recognition would be expected if the knowledge were fully conscious, the twelve different triplets in the training sequence were analyzed for learning (as measured by RT differences between deviant and standard versions of the triplet). For the positive group, eight triplets were significantly above chance (all *t*s >2.70) while four triplets were at chance (all *p*s >.20). Thus, if this knowledge were fully conscious, people should recognize these eight triplets correctly, and guess correctly half of the remaining four triplets. Thus, people’s expected performance on the recognition test would be (8*1+4*.5)/12 = 83%. In fact the upper limit of the confidence interval on people’s recognition performance is 56%, substantially below the 83% expected on the hypothesis of completely accessible conscious knowledge.

As before, 83% defines an upper limit in recognition performance expected on the theory that all knowledge is conscious because there could be random noise in any given recognition judgment [56]. A Bayes Factor pitting the theory that recognition performance could be any value from 50% to 83% with equal probability against the null that recognition is 50%, was 2.46, indicating no sensitivity [56]. However, this Bayes Factor assumes that after 15 blocks of training there could still be close to 100% noise in recognition even though the knowledge was all conscious. In fact, the noise in recognition is small compared to priming effects [56] (the authors in [56] took recognition to be conscious). If we assume that the maximum of the noise can be as high as 85% to allow recognition performance as low as 0.5+ (0.83–0.5) * (1–.85) = 55% (but no lower) when knowledge is conscious, we can represent the theory of complete conscious knowledge as a uniform between 55% and 83%. The Bayes Factor is.28 indicating evidence against the theory of complete conscious knowledge as compared to the null hypothesis of no conscious knowledge. While we reject the null hypothesis in this case for other reasons (because of finding conscious knowledge with the more focused structural knowledge attributions), if the overall recognition data more closely fit the null hypothesis rather than the theory of complete conscious structural knowledge, it is reasonable to assume partial rather than full conscious knowledge. Whether or not we reject the hypothesis of complete conscious knowledge thus depends on whether we think the noise could be as high as 85% or 100% if knowledge were fully conscious. Thus, unlike the case of learning the colour regularity, whether the evidence is consistent with the theory of complete conscious knowledge depends on just how bad one estimates conscious recognition of eight triplets might be, after extended learning. In this case, Bayes helps us appreciate why the data are not quite conclusive, but what reasonable assumptions based on prior literature would enable firmer decisions. See [32], [55] for further discussion of the use of Bayes Factors.

For the negative group, seven triplets were significantly above chance (all *t*s >2.00). Thus, if this knowledge were fully conscious, people should recognize these seven correctly, and guess the others. Thus, people’s expected performance on the recognition test would be (7*1+5*.5)/12 = 79%. In fact the upper limit of the confidence interval on people’s recognition performance is 55%, substantially below the 79% expected on the hypothesis of completely conscious knowledge.

As before, 79% defines an upper limit expected on the theory that all knowledge is conscious because of possible random noise in recognition. Pitting the theory that recognition might be equally likely any value between 50% and 79% against the null that it is 50% produces a Bayes Factor of.83, so neither of these theories is strongly distinguished. However, as before, representing the theory that all knowledge is conscious as a uniform between 55% and 79% (i.e., the noise up to 85%) recognition performance against the null gives a Bayes Factor of.01, very strong evidence against the theory that all knowledge is conscious.

In sum, the Bayesian analysis suggested that conscious knowledge in the positive group was very limited and the knowledge in both groups was largely unconscious. Consistently, neither overall recognition performance nor recognition performance based on rules or memory attributions differed significantly between the two groups, *t* (74) = .60, *p* = .55, *t* (71) = .75, *p* = .46, respectively.

### Discussion

In Experiment 1, both mood and picture ratings showed that there was a mood difference between the negative and positive groups after mood induction, suggesting that the music successfully induced positive and negative affective states. Participants in both affective groups responded to the standard stimuli faster than the shape-deviant and color-deviant ones, revealing that participants acquired both regularities, at least by the end of training. Consistent with the notion that relational processing is impaired by negative rather than positive moods [1]–[3], the negative group learned the shape regularity worse than the positive group, at least by the end of training. This finding provides evidence of the relevance of the affect-as-information hypothesis to sequence learning.

There was not clear evidence to assess the effect of mood on learning the colour regularity. Opposing predictions could be made about any possible effect of mood on learning the colour regularity. The colour regularity involved minimal integration over different time steps, as colour predicted the shape on the immediately following trial. Thus, it could be argued that people in the negative group should learn better than those in the positive group [31]. Conversely, the broaden-and-build theory [57], [58], which claims that positive emotions broaden the scope of attention, would imply that the positive group would pay more attention than the negative group to the task-irrelevant colour information and thus learn this information better than the negative group. As shown by the Bayesian analysis, the current data did not decisively determine the balance of these two possible processes.

Participants were at chance in recognizing the colour regularity, indicating that the colour regularity was unconsciously acquired. Only participants in the positive group performed above chance in the recognition test for the shape regularity when their recognition was based on rules or memory, suggesting they acquired some conscious knowledge. However, the recognition performance in both groups was considerably lower than that expected if their knowledge had been fully conscious, the Bayesian analysis was thereby able to show that the knowledge about shape regularity was largely unconscious or implicit.

A difference in learning the shape regularity between positive and negative groups was observed in the last six blocks but not the first six blocks of training. We have no special explanation for this, other than modulations of learning might be greater with greater amounts of learning. Still, we would like the support for the affect-as-information theory to be unambiguous. Experiment 2 aimed to strengthen the mood manipulation so as to produce the mood effect in only six blocks of training.

## Experiment 2

In Experiment 2, the training phase was shortened from 12 to six blocks and the mood induction was repeated after the second and fourth block to produce a more sustained mood difference. Further, one ambiguity in interpreting the results of Experiment 1 is whether the negative mood hindered learning or a positive mood helped. Thus, a neutral condition was added to explore whether implicit learning was influenced by negative or positive affects, or both. Finally, the stimuli were also changed from coloured shapes to grey shapes against different colour backgrounds to possibly highlight the colour feature and increase learning of the colour regularity. In sum, the main aim of Experiment 2 was to obtain a mood effect on learning the shape regularity, after only six blocks of training, to provide further support for the affect-as-information hypothesis [59] as applied to learning.

### Participants

One hundred and fifteen undergraduate students (56 male, 59 female) took part in the experiment. None of them had previously taken part in any implicit learning experiment. They were assigned to three groups (positive, *n* = 37; negative, *n* = 37; neutral, *n* = 41). As in Experiment 1, participants were excluded if their error proportions were greater than.15. Data from one participant in the positive group (*M* = .28), one participant in the negative group (*M* = .20), and four participants in the neutral group (*M* = .20, *SD* = .04) were excluded. All participants were tested between 9 and 11 a.m. or between 2 and 5 p.m., which was counterbalanced between the three groups.

### Materials and Procedure

For the neutral mood induction, participants were asked to read English-Chinese bilingual material about Canada, validated as neutral by previous research [11]. The materials in the SRT task were similar to those in Experiment 1 except that the stimuli were different grey shapes against different color backgrounds. The shape sequence followed the shape regularity while the color background sequence followed the color regularity.

The procedure was identical to Experiment 1 except that there were six blocks in the training phase and the shorter 5-minute mood induction was repeated after the second and fourth blocks, respectively. The repetition was about five minutes after the preceding mood induction. Previous research [60] has found that similar mood inductions could give equivalent mood effects for periods as long as five minutes. We did not ask participants to rate their mood before the repetition of the mood induction. The third and fourth mood ratings were reported after the two shorter mood inductions separately. As in Experiment 1, after each SRT block, participants were asked to rate the valence of two neutral pictures. Sixteen neutral pictures were randomly assigned to the eight different time points separately for each participant.

### Results

**Was the mood induction successful?** The first mood test was before the initial mood induction and the second one was immediately after the mood induction. To explore whether the mood manipulation was successful, an ANOVA on valence ratings with mood test (first vs. second) as a within-subject variable and group (positive vs. neutral vs. negative) as a between-subjects variable was conducted. It revealed a significant group effect, *F* (2, 106) = 12.67, *p*<.001, *η* ***_p_*** ^2^ = .19, a significant mood test effect, *F* (1, 106) = 4.02, *p*<.05, *η* ***_p_*** ^2^ = .04, and a significant interaction, *F* (2, 106) = 23.99, *p*<.001, *η* ***_p_*** ^2^ = .31. At the onset, there was no difference among the three groups (all *p*s >.25). Thereafter, negative mood inductions decreased ratings, *t* (35) = 6.18, *p*<.001, *d* = 1.04, while positive inductions increased positive ratings, *t* (35) = −3.91, *p*<.001, *d* = .66, but neutral inductions did not influence ratings, *t* (36) = −.96, *p* = .34, suggesting that the music changed people’s mood successfully. Importantly, after the mood inductions participants in the negative group had a valence significantly below neutral, *t* (35) = −3.87, *p*<.001, *d* = .65, and participants in the positive and neutral group had a valence significantly above neutral, *t* (35) = 7.76, *p*<.001, *d* = 1.31, *t* (36) = 3.60, *p*<.001, *d* = .60.

The mood ratings at the last three time points were given after the initial mood induction. To test whether there was a mood difference throughout training, an ANOVA with the last three mood tests as a within-subject variable and group (positive vs. neutral vs. negative) as a between-subjects variable was conducted. It revealed only a significant group effect, *F* (2, 106) = 30.41, *p*<.001, *η* ***_p_*** ^2^ = .37. The positive group reported higher positive ratings than the neutral group, *t* (71) = 2.76, *p*<.01, *d* = .64, while the neutral group reported higher positive ratings than the negative group, *t* (70) = 8.40, *p*<.001, *d* = 1.97, suggesting that the mood difference among the three groups existed throughout the training phase.

Similarly, the first picture rating was before the initial music induction and the second one was after the music induction. To further check the effect of the mood manipulation, an ANOVA on ratings with picture test (first vs. second) as a within-subject variable and group (positive vs. neutral vs. negative) as a between-subjects variable revealed a significant picture test effect, *F* (1, 106) = 17.11, *p*<.001, *η* ***_p_*** ^2^ = .14, which was qualified by the interaction of picture test by group, *F* (2, 106) = 6.47, *p*<.01, *η* ***_p_*** ^2^ = .11. Initially, there was no difference on the valence ratings of pictures among the three groups (all *p*s >.13). After the mood induction, the valence ratings of pictures were higher in the positive and neutral groups than in the negative group, *t* (70) = 2.95, *p*<.01, *d* = .70, *t* (71) = 2.65, *p* = .01, *d* = .62, respectively, while there were no difference between the positive and neutral groups, *t* (71) = .38, *p* = .71. The significant difference between negative and positive groups was consistent with the results of SAM test, but the results for the neutral group were not. It is surprising that there was a significant increase on ratings at time 2 for the neutral group, perhaps a mere exposure effect, suggesting that the picture rating task may have limitations for measuring mood compared with the more direct SAM test.

Picture ratings at the last seven time points were all after the initial mood induction and the ratings at time 4 and 6 were before the second and third shorter 5-minute mood inductions separately. To test whether the mood manipulation was effective, we first compared the ratings at time 1 to the ratings at time 4 and at time 6, separately for each group. The former was significantly lower than the later two for the positive group, t (35) = −2.58, p<.05, d = −.44, t (35) = −2.69, p<.05, d = −.45, respectively, but there were no significant differences between the former and later two for the neutral and negative groups (all *p*s >.05). To further test the effect of induced mood throughout training, an ANOVA on ratings of picture tests after the mood induction (last seven) as a within-subject variable and group (positive vs. neutral vs. negative) as a between-subjects variable revealed a significant group effect, *F* (2, 106) = 6.23, *p*<.01, *η* ***_p_*** ^2^ = .11. Consistently, the positive and neutral groups reported higher ratings than the negative group, *t* (70) = 3.20, *p*<.01, *d* = .75, *t* (71) = 2.89, *p*<.01, *d* = .68, respectively, but there was no difference between the positive and neutral groups, *t* (71) = .17, *p* = .86. The main effect of picture test also reached significance, *F* (6, 636) = 4.19, *p*<.05, *η* ***_p_*** ^2^ = .04. However, the interaction of picture test by group was not significant, *F* (6, 636) = .84, *p* = .61, consistent with the effect of the induced mood difference among the three groups not varying with training.

**Did mood influence learning?** The mean RTs for all shape-deviant, standard, and colour-deviant trials with correct responses were 795.95 (*SD* = 143.00), 748.62 (*SD* = 130.47), 716.77 (*SD* = 122.29), respectively, for all participants. As in Experiment 1, trials with RTs greater than 2,000 milliseconds were excluded, which lead to 2.04%, 0.32% and 2.26% of the trials being removed in the negative, neutral and positive groups, respectively.

For the shape regularity, negative, neutral and positive groups all had a learning effect significantly above zero, *t* (35) = 3.95, *p*<.001, *d* = .67, *t* (36) = 8.20, *p*<.001, *d* = 1.37, *t* (35) = 5.67, *p*<.001, *d* = .96, respectively. Importantly, one-way ANOVA on learning scores with group (positive vs. neutral vs. negative) as a between-subjects revealed a significant group effect, *F* (2, 106) = 3.14, *p*<.05. The learning effect for the positive and neutral group was significantly greater than for the negative group, *t* (70) = 2.07, *p*<.05, *d* = .49, *t* (71) = 2.40, *p*<.05, *d* = .56, respectively.

For the colour regularity, negative, neutral and positive groups all had a learning effect significantly below zero, *t* (35) = −3.36, *p*<.01, *d* = −.57, *t* (36) = −5.58, *p*<.001, *d* = −.93, *t* (35) = −4.91, *p*<.001, *d* = −.83, respectively, which is consistent with the results in the first half in Experiment 1. That is the pre-existing biases as shown in Experiment 1 were not over-ridden by training in six blocks in either Experiment 1 or 2. Importantly, one-way ANOVA on learning scores with group (positive vs. neutral vs. negative) as a between-subjects revealed no significant effect, *F* (2, 106) = .43, *p* = .65. That is, no learning differences were observed for the colour regularity among the three groups. Since no knowledge about the colour regularity was acquired by either group in Experiment 2, we did not further address whether people were consciously aware of the colour regularity.

**Were people consciously aware of the shape regularity?** If participants consciously learned the shape regularity, they would perform better than chance in the recognition for the shape regularity, especially when the recognition was based on rules or memory. For recognizing the shape regularity, participants in the positive and neutral groups performed significantly or marginally significantly above chance level, *t* (35) = 4.12, *p*<.001, *d* = .70, *t* (36) = 2.01, *p* = .053, *d* = .33, respectively, but participants in the negative group performed at chance, *t* (35) = .75, *p* = .46.

As can be seen from Table 4, participants based their judgment on both conscious and unconscious knowledge. An ANOVA on proportions with attribution (guess or random vs. intuition vs. rules or memory) as a within-subject variable and group (negative vs. neutral vs. positive) as a between-subjects revealed only a significant attribution effect, *F* (2, 212) = 21.18, *p*<.001, *η* ***_p_*** ^2^ = .17. Participants attributed to rules or memory and intuition similarly, *t* (108) = 1.04, *p* = .30, but both were greater than proportions for guess or random, *t* (108) = 7.64, *p*<.05, *d* = .74, *t* (108) = 5.38, *p*<.001, *d* = .52. Recognition was compared to chance for each combination of mood condition and attribution type. Participants in the positive and neutral groups performed above chance when they gave rules or memory attributions, *t* (34) = 2.21, *p*<.05, *d* = .38, *t* (31) = 3.85, *p* = .001, *d* = .69, respectively, but performed at chance when they gave other attributions (all *p*s >.18). Participants in the negative group performed at chance for each attribution (all *p*s >.53). The results were partially consistent with the results of Experiment 1 and indicated that positive and neutral participants were aware of some learned knowledge.

However, when expected recognition performance was estimated in the same way as for Experiment 1, the estimate given the acquired knowledge was completely conscious was 79% for the positive group, 87.5% for the neutral group and 58% for the negative group. Note in all cases, the upper limits of the confidence intervals of recognition performance (55%, 55% and 53%, respectively) are substantially below these expectations, indicating that the knowledge was largely unconscious.

Further, for the positive group, the Bayes Factor comparing the theory represented by a uniform expectation between 55% and 79% (i.e., the noise up to 85%) was.21, evidence against the theory that all knowledge was conscious. The corresponding Bayes Factors for the neutral and negative groups were.00 and.17, respectively, the latter also providing evidence against the theory that all knowledge was conscious These Bayes Factors indicate that evidence strongly supports the null over the hypothesis of fully conscious knowledge. This is consistent with there being some conscious knowledge, i.e. with the null being only approximately true (cf [61], p 233); that is, we cannot conclude from the Bayesian analysis that all knowledge was unconscious. Support for the theory that all knowledge is conscious is nonetheless low because the theory allows any amount of knowledge to be expressed over a large range, and the theory is punished for this vagueness.

Therefore, as in Experiment 1, the Bayesian analysis suggested that the knowledge acquired in all the groups was largely unconscious. Consistently, one way ANOVA on either overall recognition performance or recognition performance for only rules or memory attribution with group (negative vs. neutral vs. positive) as between-subjects revealed no significant group effect among the three groups (both *ps >*.11).

### Discussion

After the mood induction, mood ratings of the positive group were higher than the neutral group, while mood ratings of the neutral group were higher than the negative group, suggesting the mood inductions were effective. However, unlike the mood ratings, picture ratings of both positive and neutral groups were higher than negative group, but there was no difference between the positive and neutral groups. People may have a baseline positive mood. Indeed, initially, mood ratings in the three groups were slightly positive compared with the neutral point, i.e. a rating of 5 (*t* (35) = 4.35, *p*<.001, *d* = .74, *t* (36) = 3.86, *p*<.001, *d* = .64, *t* (35) = 2.40, *p*<.05, *d* = .41, respectively). After the mood induction, both mood ratings and picture ratings in the neutral group were also more positive than the neutral point (*t* (36) = 3.60, *p* = .001, *d* = .60, *t* (36) = 6.22, *p*<.001, *d* = 1.04, respectively). This is consistent with previous research, suggesting that neutrally treated participants are in fact slightly positive [62], [63]. Thus, future research might use a neutral induction that actually leaves people feeling neutral in order to more sensitively explore the effect of positive mood on implicit learning.

Participants in the three groups responded to the standard stimuli faster than the shape-deviant stimuli indicating that all participants acquired the shape regularity within six blocks. Importantly, the mood induction was repeated after every two blocks throughout training in this experiment. Unlike in Experiment 1, now the mood effect emerged within six blocks of training: The positive and neutral group learned more than the negative group of the shape regularity. Thus we can conclude that negative mood can impair implicit learning. Given the difference between the positive and neutral group was non-significant, we cannot yet conclude that a positive mood facilitates implicit learning.

As in Experiment 1, recognition of the shape regularity was above chance only when participants in the positive and neutral group gave rules or memory attributions, indicating the acquisition of some conscious knowledge. However, the performance was considerably below the performance which would be expected if all acquired knowledge could be intentionally consciously retrieved. Thus, while there was some detectable conscious recognition in the two groups, learning appeared to be largely implicit or unconscious in all groups.

## General Discussion

In two experiments, we explored the effect of negative and positive affects on implicit learning of complex sequences with the SRT task. The results of both experiments showed that negative affect reduced the acquisition of a second-order regularity, i.e. a regularity that required integrating over past trials, providing evidence for the affect-as-information hypothesis [59]. The affect-as-information hypothesis suggests that a positive mood is associated with reliance on relational, integrative information, while a negative mood leads to detailed processing of only recent information [1]–[3]. Our finding is in principle consistent with previous research [11], [64] in which negative affect focuses attention on recent events.

One prior study did not find an effect of induced mood on a second-order conditional sequence [29]. They do not report raw learning scores, but a fairly complex derived measure, so it is hard to assess the sensitivity of [29]; in the absence of an assessment of its sensitivity, nothing follows from their non-significant result. Further, the mood manipulation was very different between the two studies: [29] used 50 affective photos displayed for six and half minutes to induce the different moods before the SRT task and they did not strengthen it during the training phase; we used music pieces of ten minutes to induce the different moods before the SRT task and repeated a short mood induction of five minutes one and two times in Experiments 1 and 2, respectively.

Previous research showed superior performance for induced negative rather than positive moods on an artificial grammar learning task [29]. A key structure learned in artificial grammar learning is bigrams, i.e. the extent to which one letter directly predicts the immediately following letter. However, we failed to find an effect of negative mood on the learning of the first-order sequence in our SRT experiment. This may be because the first order conditional sequence was predicted by the task-irrelevant rather than target feature and the colour-deviant stimuli were not fully counterbalanced so longer training was required to achieve positive learning scores in the present study. In addition, the broaden-and-build theory [57], [58] suggests that the positive group would pay more attention than the negative group to the task-irrelevant colour information. Thus, the two contradictory tendencies may have cancelled the effect of mood on a first order conditional sequence (the colour regularity). Future research should investigate whether more global structures in artificial grammar learning are in fact learned better when positive rather than negative moods are induced, and more local structures in the SRT task are learned better when negative rather than positive moods are induced. Future research could also explore the cross cultural sensitivity of mood effects on implicit learning of SOC sequences. [65] found East Asians acquired more accurate unconscious knowledge of more global rather than local structures relative to Westerners; thus, how mood affects the implicit learning of SOC sequences in people of different cultures remains an open question.

An important question is the extent to which our conclusions apply to distinctively implicit learning. Interestingly, participants in the positive or neutral group showed detectable conscious knowledge in both experiments, while participants in the negative group did not. Recent data show that positive affect can both enhance visual awareness in patients suffering from visual neglect [66], and reduce attentional blink in healthy individuals [67], indicating that positive affect can enhance awareness. Future research, with a more sensitive design, could explore if positive affect enhances the transition from unconscious to conscious knowledge. Importantly, while there was detectable conscious knowledge of the shape regularity in the positive or neutral group, it fell short of that expected if all the knowledge was conscious. Further, even allowing for the fact that the SRT and recognition tests may simply differ in test sensitivity to a common conscious knowledge base [34], [39], a Bayesian analysis showed that the null hypothesis had more support than the theory that all knowledge was conscious, especially in Experiment 2. Thus, in our data, there is an effect of mood on *implicit* learning rather than *explicit* learning (contrast [68], for the argument that all knowledge is conscious). Momentary mood may modulate not necessarily the total amount of unconscious knowledge but what it consists of – over what time scale it integrates. Future research should test this possibility.

We should also note some limitations of the present study. First, although we include a neutral mood condition in Experiment 2, it turned out neutral participants performed very similarly to positive people on both picture ratings and learning scores. Future research, however, should test whether there is a difference between positive and neutral states. Second, the arousal dimension of affect has been found to facilitate a tendency for positive mood to increase relational processing [69]. Further studies should compare influence of moods that varied independently in valence and arousal on implicit learning (see [60], for a possible method). Third, the mood inductions were fairly clearly mood inductions, and according to e.g. [5], when people know why their mood has changed, mood has less impact on cognition. Despite this, we did achieve an effect of mood on learning, though future research may wish to change mood more subtly.

In summary, we have shown that even the information we acquire without awareness, and thus our resultant capacities and abilities, are modulated by momentary feeling states. The interaction between cognition and emotion runs deeper than previously shown.
